# Sugar Depletion in Korean Pine Infected with *Cronartium ribicola*, Alongside Metabolic Reprogramming by the T6P–Tre Signaling Module

**DOI:** 10.3390/ijms27219441

**Published:** 2026-10-23

**Authors:** Jianan Wang, Bing Wang, Qi Yu, Ruizhi Zhang, Bin Tan, Zifei Feng, Feng Wang, Danlei Li

**Affiliations:** 1 Key Laboratory of Alien Forest Pest Detection and Control-Heilongjiang Province, College of Forestry, Northeast Forestry University, Harbin 150040, China; wangjianan@nefu.edu.cn (J.W.); wangbing@nefu.edu.cn (B.W.); yinr@nefu.edu.cn (Q.Y.); zhangruizhi@nefu.edu.cn (R.Z.); tanbin@nefu.edu.cn (B.T.); fengzigei@nefu.edu.cn (Z.F.); 2 Key Laboratory of Sustainable Forest Ecosystem Management-Ministry of Education, Northeast Forestry University, Harbin 150040, China

**Keywords:** biotrophic pathogen, sugar metabolism, aecia, *Pinus koraiensis*, *Cronartium ribicola*, T6P, trehalose

## Abstract

In diseased Korean pine trees (*Pinus koraiensis*), *Cronartium ribicola* develops aecia only in the bole, where sugar is initially sufficient. However, the molecular mechanisms underlying sugar depletion in diseased Korean pine trees remain unelucidated, hindering sugar-targeted control strategies. Focusing on host responses, this study integrated metabolomics, transcriptomics, and targeted glycomics based on spatial partitioning to investigate sugar metabolic reprogramming in the bole of diseased pines. Multi-omics revealed severe sugar depletion in the blister area, which was significantly correlated with relative *C. ribicola* DNA level. However, the transcription of sugar synthesis genes was upregulated, while sugar degradation genes were downregulated, indicating decoupling between sugar metabolism and transcription. Concurrently, T6P–Tre-centered reprogramming was identified: *Pktppd* and *Pktpp4*, encoding enzymes that convert T6P into Tre, were downregulated, leading to elevated T6P and reduced Tre levels. This T6P–Tre shift likely acts as a signal to activate genes related to sugar accumulation in response to sugar depletion, thereby explaining the observed transcriptional-metabolic decoupling. Targeting the T6P–Tre module to modulate host sugar allocation and restrict sugar availability to the pathogen may offer a novel strategy for the targeted control of Korean pine blister rust (KPBR).

## 1. Introduction

When the biotrophic pathogen *Cronartium ribicola* infects Korean pine (*Pinus koraiensis*) for 2–3 years, it is presumed to rely on a continuous nutrient supply from the host [1]. Meanwhile, numerous blister-like aecia develop on the bole [2]. Controlling aecia development is a key step in curbing outbreaks of Korean pine blister rust (KPBR): after aeciospores infect alternate hosts, the mass production of urediniospores drives the *C. ribicola* population’s exponential growth [3]. Preventing aecia development can break the infection cycle before the pathogen population expands, thereby achieving KPBR control [4]. Sugar from Korean pine is considered a major source of carbon and energy for the biotrophic pathogen *C. ribicola* during aeciospore production. Successful carbohydrate uptake from the host is essential for obligate parasites, particularly rust [5]. Targeting the sugar supply in Korean pine to inhibit aecia development is a key strategy for KPBR control. However, the molecular mechanisms driving host sugar depletion in diseased Korean pine remain poorly understood, limiting sugar-targeted disease control.

Korean pine stores two categories of carbon compounds in its bole: structural components that provide mechanical support, and non-structural carbohydrates (NSCs), including soluble sugars and starch [6]. This study focuses on soluble sugars in NSCs, which are the most direct and rapidly metabolized carbon source for pathogens [7,8,9]. Aecia develop in the phloem of the Korean pine bole [10]. This tissue is responsible for both soluble sugar transport and storage [9] and, acts a carbon hub, linking the canopy to the root system. It is a key site for the supply of soluble sugars to biotrophic pathogens [11]. Research has demonstrated that mutations in the *SWEET* sucrose transporter genes can effectively confer broad-spectrum resistance in plants [12]. Therefore, blocking the supply of soluble sugars by interfering with key gene pathways involved in sugar metabolism in the bole’s blister area may effectively prevent aecia development [13]. However, the relationship between transcriptional regulation and metabolic flux in host sugar metabolism within the blister area remains unclear. The mechanisms by which *C. ribicola* influences sugar reallocation in Korean pine, as well as the key factors mediating this process, remain to be elucidated. Answering this question requires integrated transcriptomic and metabolomic analyses rather than single-omics inferences.

Trehalose (Tre) is a non-reducing disaccharide that functions as a storage carbohydrate, a transport sugar, and a signaling molecule [14,15]. It is synthesized in two steps: Trehalose-6-phosphate synthase (TPS) converts UDP–glucose and glucose-6-phosphate (G6P) to trehalose-6-phosphate (T6P), which is then dephosphorylated by trehalose-6-phosphate phosphatase (TPP) to yield Tre [16,17]. Beyond serving as a metabolic precursor of Tre, T6P is a key sugar signaling molecule that reflects sugar availability. Elevated levels of T6P promote sugar biosynthesis while suppressing sugar degradation [18,19,20]. Given that plants typically accumulate only low levels of Tre [21,22], plants appear to have evolved a sophisticated system for the synthesis and breakdown of T6P and Tre. Tight control of T6P and Tre’ cellular concentrations, with T6P acting as a key regulatory factor in the metabolism of sugars such as sucrose [23], allows T6P and Tre to play a role in the regulation of plant metabolism and development [24,25,26,27].

Field observations show that in Korean pine infected with *C. ribicola*, aecia only develops in the bole, where sugars are initially abundant. Based on this observation, we hypothesized that pathogen-induced reprogramming of host sugar metabolism occurs in the blister area. Focusing on host responses, this study integrated transcriptomics, metabolomics, and targeted glycomics using spatially partitioned infected bole and branch tissues to characterize sugar metabolic changes in the blister area. This work aims to elucidate the transcriptional–metabolic features of host sugar metabolism in the blister area and to identify host factors associated with this state, thereby providing a theoretical basis for host sugar-targeted control of KPBR.

## 2. Results

### 2.1. Spatial Confinement of Aecia Development to the Bole

**Figure 1:**
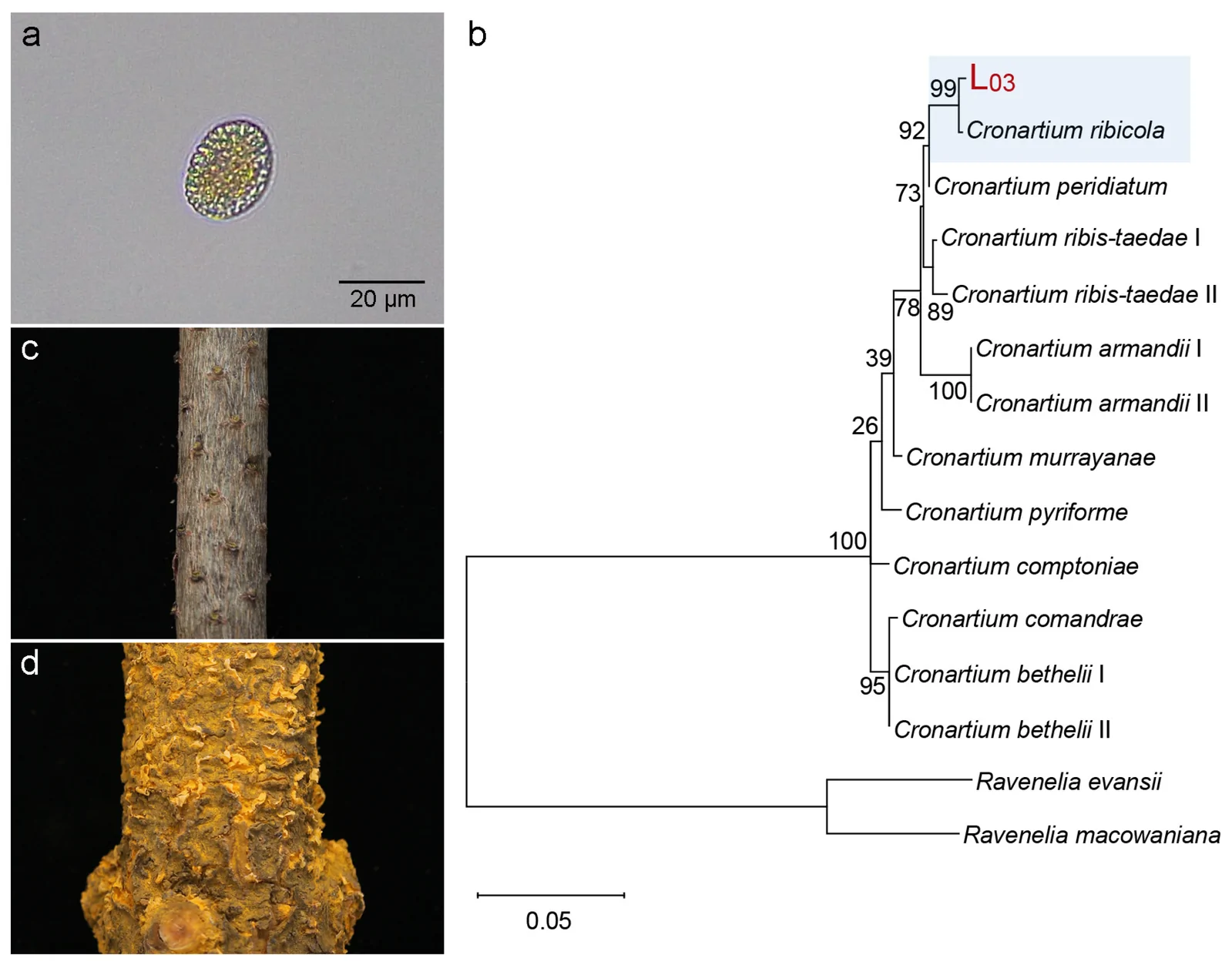
Symptoms of KPBR and identification of *C. ribicola*. (**a**) aeciospore morphology of *C. ribicola* (scale bar = 20 μm); (**b**) phylogenetic tree of *C. ribicola* based on ITS-LSU sequences; (**c**) the Korean pine branch exhibiting no obvious symptoms; (**d**) circular aeciospore-producing blisters (sori) on the bole of Korean pine 150 cm below the ground.

In three 15-year-old Korean pine plantation plots, 356 trees infected with blister rust were continuously monitored. The pathogen was identified as *C. ribicola* based on morphological characteristics (Figure 1a) and phylogenetic analysis of ITS and LSU sequences (Figure 1b). Trees exhibited a characteristic pattern: no aecia were observed on the branches of any symptomatic trees examined (Figure 1c). Aecia were primarily concentrated on the bole, arranged in a ring pattern (Figure 1d). These results indicated that aecia development is spatially restricted and primarily confined to the boles of Korean pine. The bole was designated as the “blister area”, while the branch was designated as the “non-blister area”. Healthy Korean pines were indicated as CK, with the bole and branch designated as “healthy bole” and “healthy branch”, respectively.

### 2.2. Severe Depletion of Sugar Reserves, Including Tre, in the Blister Area

**Figure 2:**
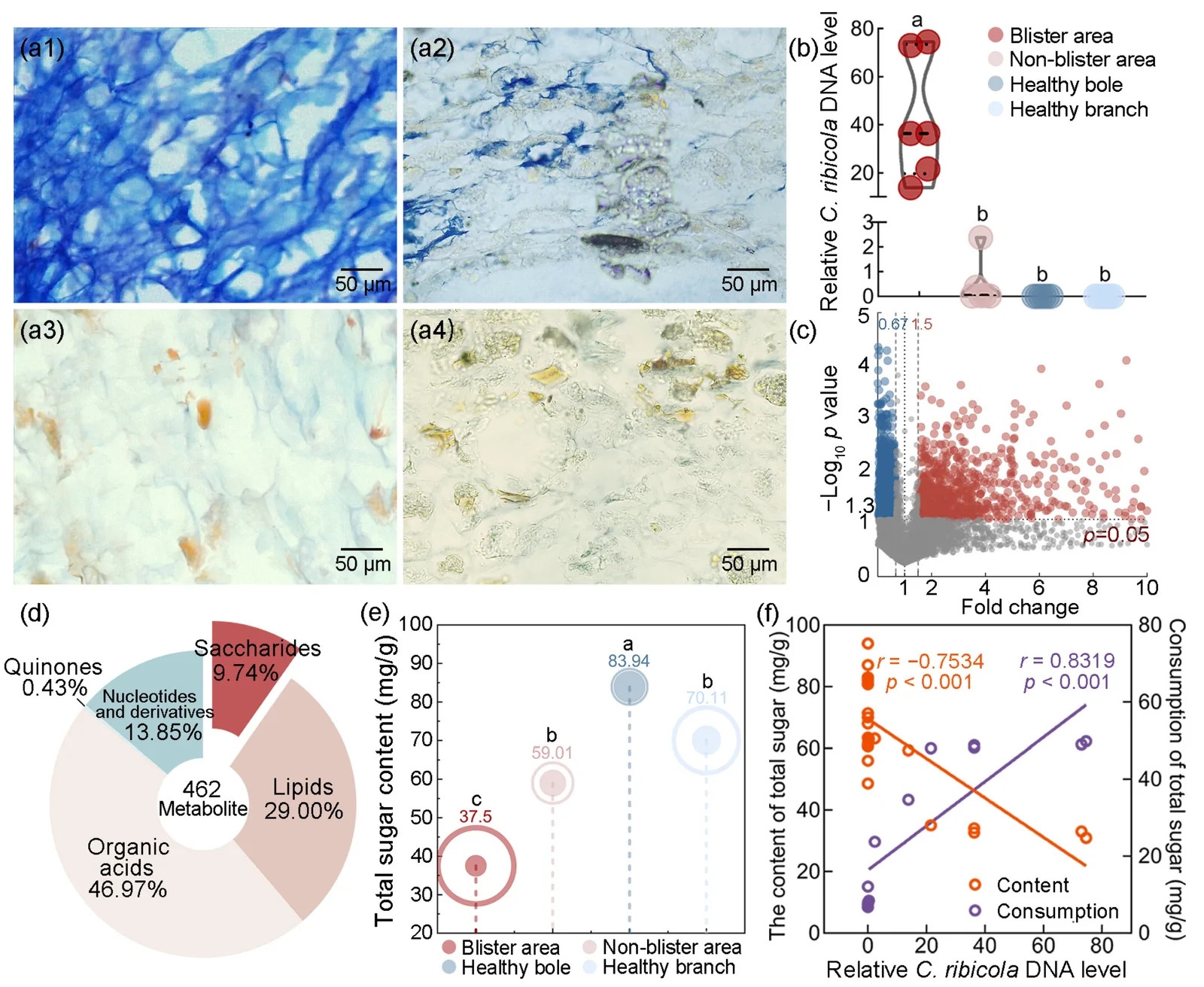
*C. ribicola* DNA level is strongly correlated with sugar depletion. Trypan blue staining showing hyphal colonization of *C. ribicola* in Korean pine tissues: (**a1**) dense hyphae in the diseased tree’s blister area; (**a2**) sparse hyphae in the diseased tree’s non-blister area; (**a3**) healthy tree bole; (**a4**) healthy tree branch; (**b**) quantification of relative *C. ribicola* DNA level in four types of tissue, as determined by qPCR; (**c**) volcano plot of DAMs in blister and non-blister areas (red dots: upregulated; blue dots: downregulated); (**d**) composition of energy supply-related DAMs between blister and non-blister areas; (**e**) determination of total sugar content in four types of tissue; (**f**) correlation analysis of relative *C. ribicola* DNA level with the content and consumption of total sugar; the lowercase letters a, b, c within figures denote statistical significance groups determined by one-way ANOVA followed by multiple comparison; different letters indicate significant differences (*p* < 0.05).

To determine and quantify the spatial distribution of *C. ribicola*, Trypan blue staining and quantitative PCR (qPCR) were performed. Trypan blue staining revealed that, although both the blister and non-blister areas of diseased Korean pines were infected by *C. ribicola*, the hyphal density was significantly higher in the blister area (average infection level 3.6 ± 0.5) than that in the non-blister area (1.4 ± 0.5). By contrast, no hyphae were observed in the corresponding areas of healthy controls (Figure 2(a1–a4)). qPCR further confirmed that the relative accumulation of *C. ribicola* DNA was significantly greater in the blister area (mean 42.61) than in the non-blister area (mean 0.48; Mann–Whitney *U* test, *p* < 0.01). *C. ribicola* DNA was not detected in any healthy controls (Figure 2b). Collectively, these results demonstrate that both colonization density and pathogen DNA level were substantially greater in the blister area than in the non-blister area.

To assess whether the spatial distribution of *C. ribicola* in the blister area correlates with metabolite distribution, untargeted metabolomics was performed. A total of 7788 metabolites were detected by untargeted metabolomics across four tissue types: the blister area and non-blister area of symptomatic Korean pines, and the corresponding “healthy bole” and “healthy branch” of healthy Korean pines. Compared with the non-blister area, the blister area of symptomatic trees exhibited 749 significantly increased metabolites and 1048 significantly decreased metabolites (*p* < 0.05; Figure 2c).

As obligate biotrophic fungi rely on the host for their energy supply, this study focused on metabolites associated with this. Of the differentially accumulated metabolites (DAMs) related to energy supply, carbohydrates accounted for 9.74% and lipids for 29.00% (Figure 2d). Sugars and lipids are the primary energy storage compounds among the metabolites associated with energy. Due to their water solubility and rapid energy release, sugars are preferentially utilized over lipids. Accordingly, the total sugar content was determined, with the result showing severe depletion in the blister area (Figure 2e). The total sugar content in the healthy bole (corresponding to the blister area; 83.94 ± 5.02 mg/g) was significantly higher than the healthy branch (corresponding to the non-blister area; 70.11 ± 9.19 mg/g; *p* < 0.05). This indicates a basipetal gradient of total sugars, with higher levels in healthy boles than in healthy branches. In symptomatic trees, however, this gradient was reversed: the total sugar content in the blister area (37.50 ± 10.78 mg/g) was significantly lower than the non-blister area of the same tree (59.01 ± 5.73 mg/g; *p* < 0.01); it was also significantly lower than the corresponding healthy tissue (*p* < 0.001). Relative to the healthy baseline, total sugar consumption in the blister area (46.44 mg/g; relative depletion rate of 55.33%) was significantly greater than the non-blister area (11.09 mg/g; 15.82%; *p* < 0.001, Cohen’s *d* = 3.45), representing an approximately 4.2-fold difference.

A correlation analysis was performed to evaluate the relationship between the total sugar levels and relative *C. ribicola* DNA level. This showed a negative correlation (Pearson’s *r* = −0.7534, *p* < 0.001, Figure 2f). In comparison, total sugar consumption exhibited a stronger positive correlation with the relative *C. ribicola* DNA level (Pearson’s *r* = 0.8319, *p* < 0.001, Figure 2f), suggesting that greater pathogen colonization was associated with more pronounced sugar depletion.

**Figure 3:**
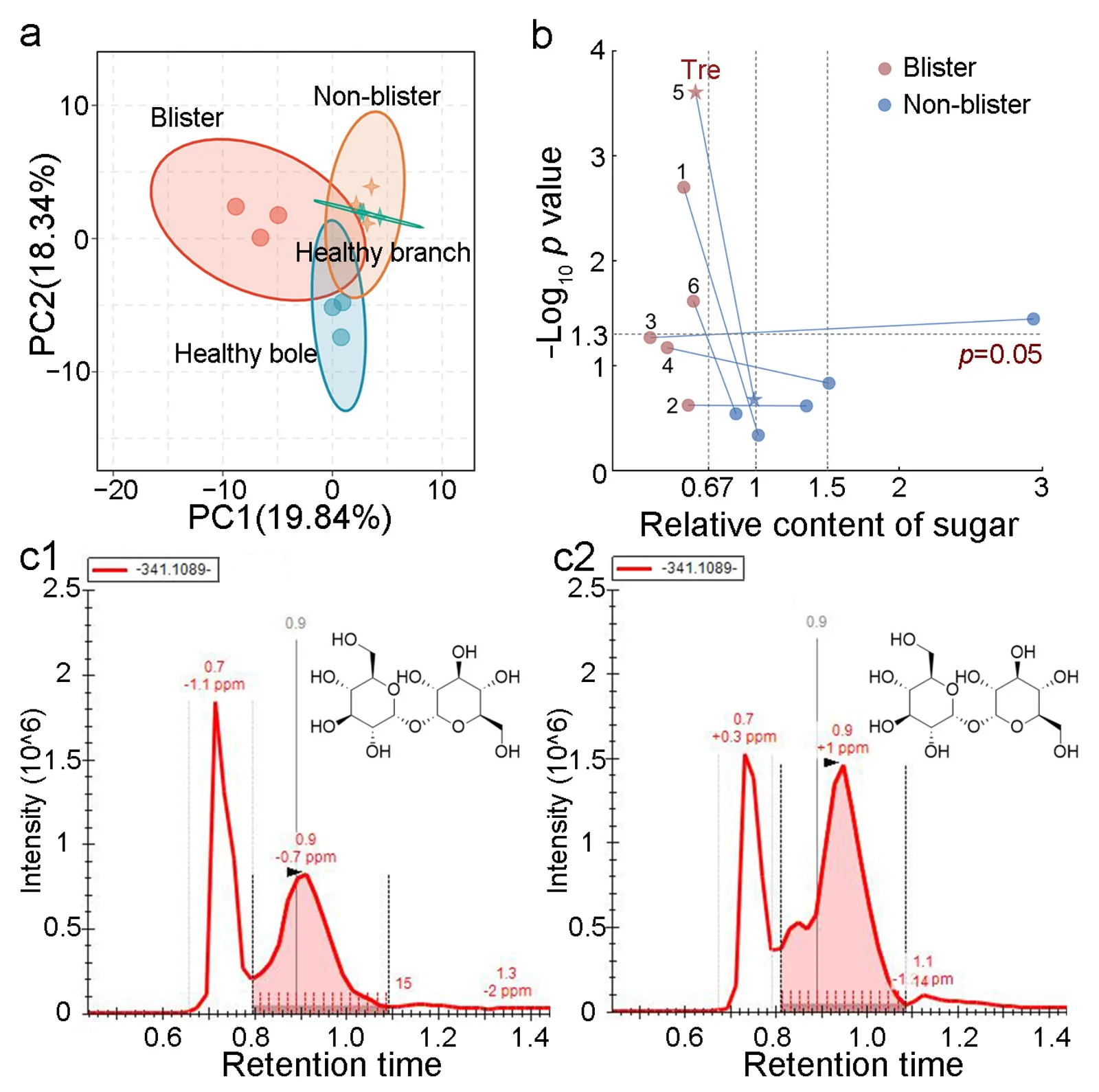
Metabolomic analysis of the blister and non-blister areas of Korean pine. (**a**) PCA of metabolic profiles for soluble sugars; (**b**) analysis of the relative content of 6 sugar compounds; 1–6 represent alpha,beta-trehalose, globotriose, gal1-3[fuc1-4]glcnacsp, UDP-D-galactose, Tre, and amylotetraose; red dots: blister area; blue dots: non-blister area; star indicate the Tre, and blue lines connect the same metabolite between blister and non-blister tissues; (**c1**) chromatogram of Tre in the blister area; (**c2**) chromatogram of Tre in the non-blister area.

Compared with structural carbohydrates (SCs), soluble sugars are mobile and metabolically accessible. They serve not only as primary energy sources but also as key modulators of sugar metabolism during host–pathogen interactions. Among the detected metabolites, 86 were soluble sugars. PERMANOVA revealed highly significant overall differences in metabolic profiles across the four groups (pseudo-*F* (3,8) = 3.297, *R*^2^ = 0.553, *p* < 0.001). PCA visualization showed that the blister area and healthy bole were completely separated along the PC1 axis, while the non-blister area and healthy branch had similar metabolic profiles (Figure 3a).

Compared with the non-blister area and healthy bole, six compounds were found to be significantly depleted in the blister area (Figure 3b). Notably, four of the six compounds were directly implicated in energy metabolism, including alpha,beta-trehalose, UDP-D-galactose, trehalose (Tre), and amylotetraose. Among these metabolites, Tre was significantly affected; notably, it serves as both a carbohydrate source and a signaling molecule. Its low abundance in the blister area was detected by using chromatographic analysis (Figure 3(c1,c2)).

These results indicate that the blister area underwent intensive sugar depletion following disease onset, leading to a marked decline in sugar reserves, notably Tre. This decline may be an important factor in sugar metabolism and may be involved in aecia development.

### 2.3. Transcriptional Reprogramming of Sugar Metabolism in the Blister Area: Enhanced Synthesis and Suppressed Degradation

**Figure 4:**
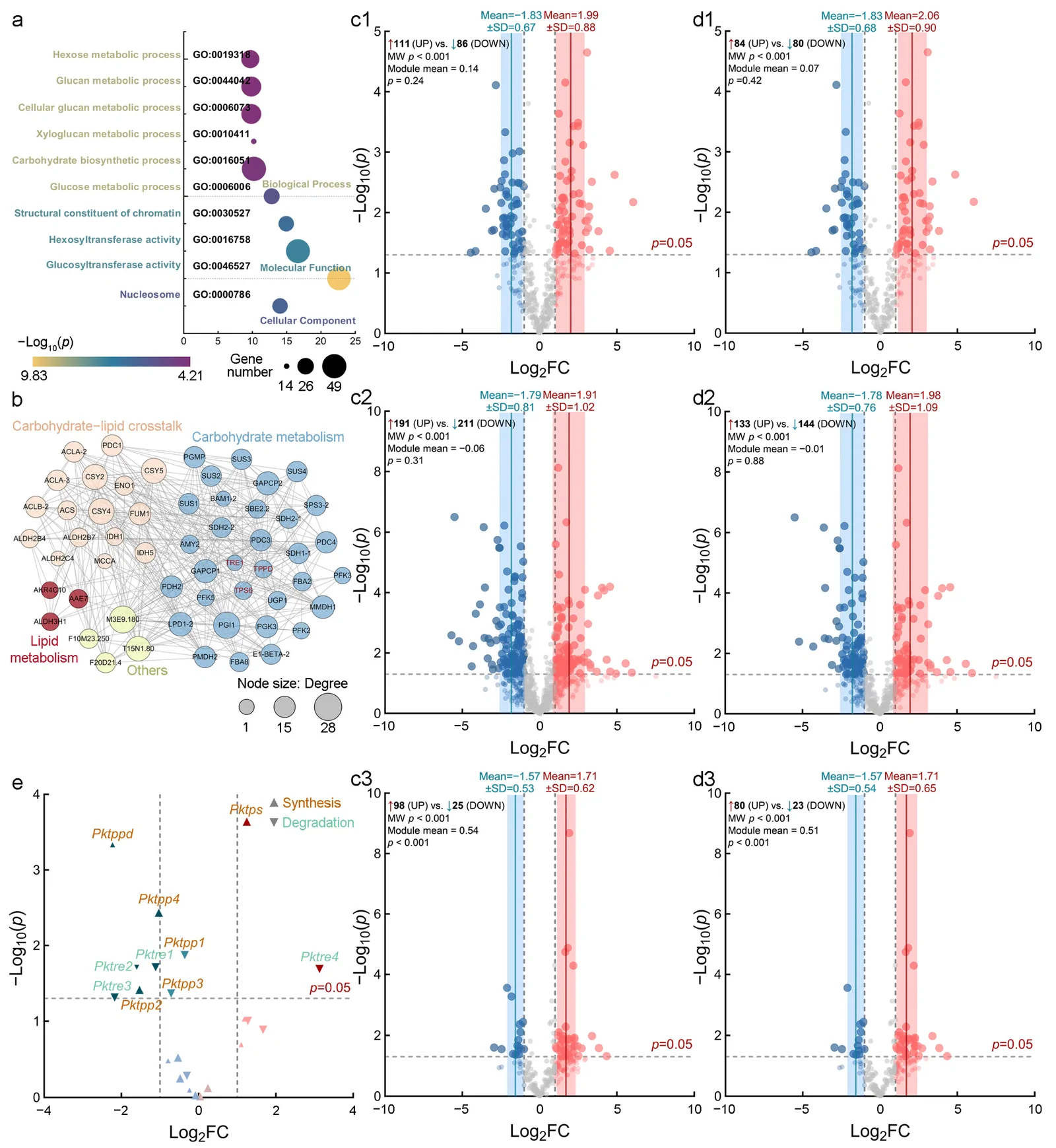
Transcriptomic reprogramming of sugar metabolism is inconsistent with severe sugar depletion. (**a**) GO enrichment analysis of DEGs between the blister and the non-blister areas; (**b**) homologous transcriptional metabolic network constructed from known *A. thaliana* regulatory pathways. Tre enzymes and various sugar metabolism enzymes are regulated coordinately; (**c1**–**c3**) volcano plots of sugar metabolism genes (|Log_2_FC| > 1): (**c1**) synthesis, (**c2**) degradation, and (**c3**) transport modules; (**d1**–**d3**) volcano plots of soluble sugar-specific genes (*p* < 0.05): (**d1**) synthesis, (**d2**) degradation, and (**d3**) transport modules; pink and blue denote upregulation and downregulation, respectively; larger dots denote significant changes; (**e**) expression of Tre metabolism genes: volcano plot of TPS/TPP (upward triangle representing synthesis module) and trehalase (TRE, downward triangle representing degradation module).

Transcriptome sequencing was performed on the same four tissue types to investigate the molecular basis of the marked sugar depletion in the blister area. High-quality sequencing data were obtained for all samples, supporting the robustness of subsequent differential expression analysis. Gene Ontology (GO) enrichment analysis showed that differentially expressed genes (DEGs) were enriched in carbohydrate metabolic process, ATP binding, and defense response (Figure 4a). Genes specifically upregulated in the blister area and involved in energy metabolism were identified. A homology-based transcriptional network reconstructed from *Arabidopsis thaliana* pathways revealed coordinated regulation of numerous sugar metabolism enzymes. The expression of TPS and TPPD (a D isoform of TPP), involved in Tre synthesis metabolism, and trehalase (TRE), involved in Tre degradation, were differentially regulated with various catalytic enzymes involved in energy metabolism (Figure 4b). These results are consistent with the metabolomic analysis.

Significant differences in global transcriptional profiles among the four tissue types were detected using PERMANOVA (pseudo-*F* (3,8) = 6.765, R^2^ = 0.717, *p* < 0.001). Among the 78,282 transcripts, 1494 genes associated with sugar metabolism were identified. These genes were assigned to three functional modules: biosynthesis (424 genes), degradation (816 genes), and transport (254 genes).

Genes related to sugar metabolism showed greater upregulation of biosynthesis and transport genes and suppression of degradation genes. To ensure greater accuracy in the transcriptomic analysis results, a four-group comparative analysis (blister/healthy bole vs. non-blister/healthy branch) was employed. At a relaxed fold-change threshold of |Log_2_FC| > 1, 111 genes in the biosynthesis module were classified as upregulated and 86 as downregulated. Genes encoding enzymes involved in cell-wall and Suc biosynthesis were predominately upregulated (Figure 4(c1)). The degradation module showed a numerical bias toward downregulation, with 191 genes classified as upregulated and 211 as downregulated (Figure 4(c2)). The transport module contained 98 upregulated and 25 downregulated genes. Among the transporter genes with significant expression changes, those encoding plasma membrane sugar efflux carriers were upregulated (Figure 4(c3)). Collectively, biosynthesis was induced, degradation was biased toward suppression, and transport was characterized by upregulated plasma membrane efflux carriers.

For soluble sugar metabolism-related genes, the results were consistent with global patterns. A total of 1113 genes annotated for soluble sugar metabolism were analyzed, including 353 genes involved in biosynthesis, 548 in degradation, and 212 in transport. Within the biosynthesis module, 51 genes were significantly upregulated and 42 were significantly downregulated (*p* < 0.05). The significantly upregulated genes were predominantly fructose-bisphosphate aldolase (FBA) and sucrose synthase (SUS) isoforms and genes encoding key gluconeogenic enzymes (Figure 4(d1)). A pronounced bias toward downregulation was observed in the degradation module, with 85 genes significantly downregulated and only 58 upregulated. The significantly downregulated genes included those encoding glycosyl hydrolases (GHs), β-glucosidases, and invertases, indicating broad suppression of carbohydrate-degrading capacity (Figure 4(d2)). In the transport module, 32 genes were significantly upregulated and 13 were downregulated. The upregulated genes primarily encoded plastid-localized sugar-phosphate translocators (Figure 4(d3)). Among the significantly upregulated genes encoding plasma membrane sugar transporters in this module, *Pkmst6* (Cluster-51999.2) showed the highest expression, with a 4.34-fold increase in transcript abundance. Collectively, sugar metabolism underwent reprogramming. This was characterized by induced biosynthesis and suppressed degradation of soluble sugars, inconsistent with the sugar depletion observed.

Twenty-two genes involved in Tre metabolism met the expression threshold for analysis, including 15 *Pktps/Pktpp* genes in the biosynthesis module and seven *Pktre* genes in the degradation module (Figure 4e, Table S1). Among the 15 *Pktps/Pktpp* genes, one *Pktps* (Cluster-124487.1) was upregulated (Log_2_FC = 1.25, *p* < 0.001). The *Pktpp* genes were generally downregulated, with *Pktppd*, *Pktpp2*, and *Pktpp4* showing significant repression (Log_2_FC = −2.23, −1.53, −1.03, *p* < 0.05). Among the seven Pktre genes, only one was significantly upregulated, whereas three were significantly downregulated (Cluster-129996.10, Log_2_FC = −1.11, *p* = 0.019; Cluster-108716.0, Log_2_FC = −1.60, *p* = 0.019; Cluster-129996.7, Log_2_FC = −2.17, *p* = 0.049), providing no evidence of transcriptional activation of Tre degradation.

### 2.4. The T6P–Tre Module Orchestrates Sugar Metabolic Reprogramming in the Blister Area

**Figure 5:**
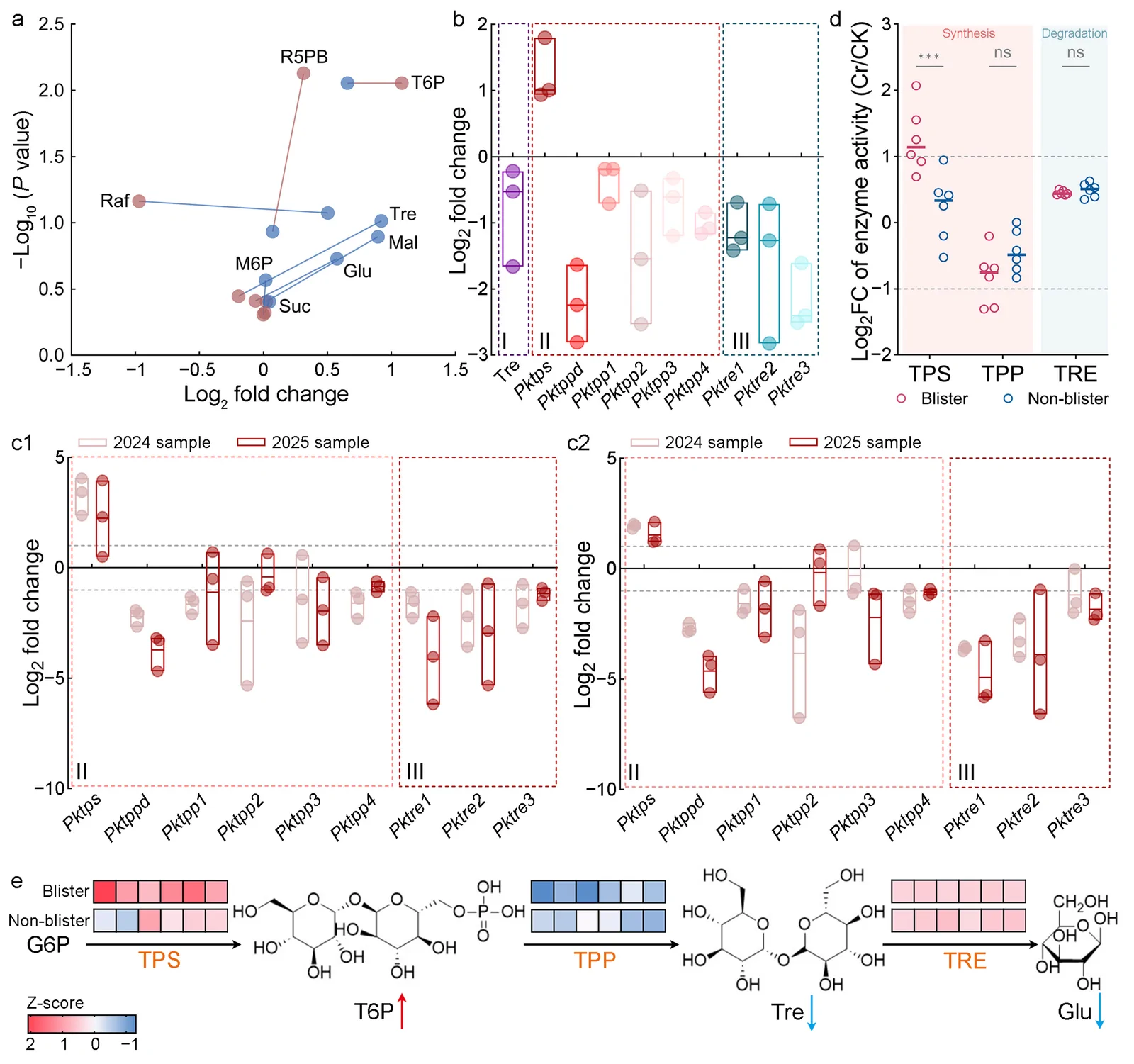
Glycomics, transcriptomics, and enzyme assays reveal metabolic–transcriptional inconsistency in the blister area. (**a**) Targeted glycomics revealed that the sugars involved in Tre metabolism were depleted in the blister area relative to the non-blister area; red dots: blister area; blue dots: non-blister area; lines connect the same metabolite between blister and non-blister tissues, pink lines indicate higher relative content in blister, and blue lines indicate higher relative content in non-blister; upstream sugars of Tre: T6P (trehalose-6-phosphate), M6P (mannose 6-phosphate), R5PB (barium D-ribose-5-phosphate); Tre: trehalose; Tre degradation products: Glu (glucose); downstream sugars of Tre: Suc (sucrose), Mal (maltose), and Raf (raffinose); (**b**) RNA-seq revealed the inconsistency between the depletion of Tre and the upregulation of Tre biosynthetic genes and downregulation of trehalase genes; I: relative content of Tre in untargeted metabolomics; II: Tre biosynthetic genes; III: trehalase genes; (**c1**) RT-qPCR of the Tre biosynthetic genes and trehalase genes using *18S rRNA* as a reference gene; (**c2**) RT-qPCR of the Tre biosynthetic genes and trehalase genes using *Actin* as a reference gene; (**d**) determination of enzyme activities involved in Tre metabolism; pink background indicates synthases, and blue background indicates degrading enzyme. *** *p* < 0.001; ns, not significant; (**e**) analysis of enzyme activities in the T6P–Tre module. The color-coded blocks represent the activity of the catalytic enzyme; red arrows indicate metabolite accumulation, and blue arrows indicate metabolite reduction.

Targeted glycomics was performed to confirm the sugar changes detected by untargeted metabolomics. Absolute quantification of 32 sugars was achieved in the blister area, non-blister area, and the corresponding tissues of healthy Korean pine, represented as CK. Among the eight sugars associated with Tre metabolism, four were lower in the blister area (Figure 5a), including Tre, glucose (Glu), maltose (Mal), and raffinose (Raf). However, T6P was significantly upregulated. Compared with the non-blister area, there was no significant change in the levels of sucrose (Suc), barium D-ribose-5-phosphate (R5PB) and mannose 6-phosphate (M6P).

Among the eight sugars, only T6P, which reflects sugar levels and serves as a precursor to Tre, was significantly increased in the blister area in response to sugar depletion (Log_2_FC = 1.08, Figure 5a). Despite this increase in T6P, Tre was depleted in the blister area compared with the non-blister area. The relative content of Glu, a hydrolysis product of Tre, was also significantly lower in the blister area than in the non-blister area. This was accompanied by a consistent reduction in Tre downstream sugars, Raf and Mal. No increase in starch content was detected in the blister area (Figure S1), indicating that the consumed sugars were not converted into starch.

Targeted glycomics revealed that the mean Tre content in the blister area (0.026 μg/mg) was less than half that in the non-blister area (0.056 μg/mg), and this depletion was consistent with the untargeted metabolomic data (FC = 0.66, Figure 5b). Tre depletion was thus confirmed across two independent platforms (Figure 5a,b).

Transcriptomic expression profile revealed reprogramming of Tre metabolism. The *Pktps* gene, which catalyzes T6P synthesis, was significantly upregulated. The expression of all *Pktpp* genes involved in the conversion of T6P into trehalose (*Pktppd*, *Pktpp1*, *Pktpp2*, *Pktpp3*, and *Pktpp4*) was downregulated, with *Pktppd*, *Pktpp2*, and *Pktpp4* showing significant downregulation. The *Pktre* genes, which encode TRE, an enzyme responsible for catalyzing the hydrolysis of Tre to Glu, exhibited significantly reduced expression relative to the non-blister area (Figure 5b).

The real-time quantitative PCR (RT-qPCR) analysis was used to validate the transcriptome data. Three reference genes were selected, among which *18S rRNA* and *Actin* were more stable than *GAPDH* (Figure S2). Therefore, normalization was performed using *18S rRNA* and *Actin*. To fully verify the reliability of the transcriptome data, two sets of samples collected in 2024 and 2025 were used, and ten genes were randomly selected for validation. The results were consistent with the transcriptome data, confirming the transcriptome’s reliability (Figure S3).

Furthermore, using the two sample sets collected in 2024 and 2025 and the two reference genes, RT-qPCR confirmed that *Pktps* was significantly upregulated in the blister area. The *Pktppd* and *Pktpp4* genes were all significantly downregulated, alongside *Pktre1*, *Pktre2* and *Pktre3* were all significantly downregulated (Figure 5(c1,c2) and Figure S4, Table S2). This expression pattern agrees with the transcriptomic data.

To determine whether TPS, TPP, and TRE were functionally active, the enzymatic activities of the three proteins were measured. The results showed that TPS activity was significantly increased in the blister area, whereas TPP and TRE activities showed no significant changes, consistent with the transcript-level results (Figure 5d).

Overall, the reduction in *Pktre* expression and unchanged TRE activity indicated that Tre degradation was not enhanced. Activation of *Pktps* and TPS promoted T6P synthesis, whereas downregulation of *Pktpp* and TPP inhibited the conversion of T6P into Tre. This led to decreased Tre levels and accounted for the concomitant accumulation of T6P (Figure 5e).

**Figure 6:**
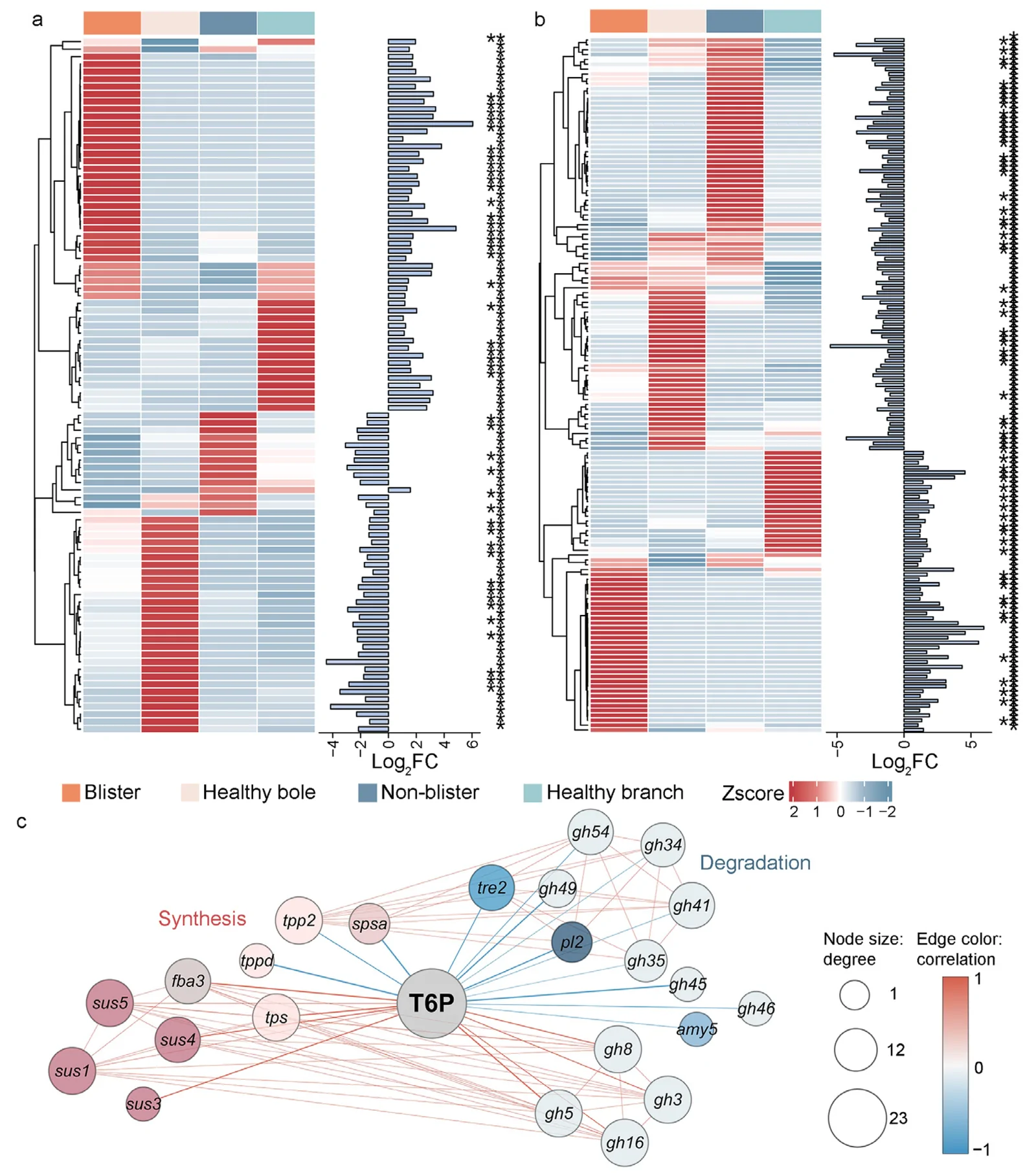
T6P-associated transcriptional reprogramming of sugar metabolism in the blister area. (**a**) expression and clustering of soluble sugar synthesis genes; (**b**) expression and clustering of soluble sugar degradation gene; (**c**) correlation analysis of T6P with sugar synthesis and degradation genes; * *p* < 0.05; ** *p* < 0.01; colored lines represent correlations: red indicates positive correlation and blue indicates negative correlation.

T6P is a key signaling molecule. Elevated levels of T6P promote sugar synthesis and inhibit sugar degradation. The expression patterns of the DEGs involved in sugar synthesis and degradation are shown in Figure 6a,b. Correlation analysis between these DEGs and T6P levels revealed that T6P was significantly correlated with nine sugar synthesis genes, of which six showed positive and three showed negative correlations. The sugar synthesis genes positively correlated with T6P included *fba*, *sus*, and *tps*. In contrast, among the 14 sugar degradation genes directly correlated with T6P, four showed positive correlations, whereas 10 exhibited negative correlations (Figure 6c, |*r*| > 0.8, *p* < 0.05). The sugar degradation genes negatively correlated with T6P included *tre*, and, predominantly *gh* genes. These results align with the established role of elevated T6P in promoting sugar synthesis while suppressing sugar degradation.

## 3. Discussion

This study focused on host responses, identifying that severe sugar depletion in the blister area is significantly correlated with relative level of *C. ribicola* DNA. Unexpectedly, transcriptomic profiling revealed the upregulation of sugar synthesis genes and downregulation of sugar degradation genes, despite this depletion. This transcriptomic-metabolomic inconsistency indicates that the transcriptional activation of sugar synthesis did not lead to sugar accumulation, revealing a host sugar status that is not simply mirrored by transcription. We therefore propose a “synthesis–accumulation decoupling” model to describe this contradiction. Concurrently, a T6P–Tre-centered reprogramming was identified: downregulation of the *Pktpp* genes, encoding enzymes that convert T6P into Tre, was associated with elevated T6P and reduced Tre levels. Given the signaling role of T6P, this shift likely activates genes related to sugar accumulation in response to sugar depletion, explaining the observed transcriptomic–metabolomic inconsistency. Together, these findings suggest that the T6P–Tre module may be a key host regulatory node associated with sugar depletion during aecia development, providing a molecular framework for the sugar-targeted control of KPBR.

The “synthesis–accumulation decoupling” model indicates that upregulated sugar synthesis at the transcriptional level in the host blister area does not result in sugar accumulation at the metabolic level. It was hypothesized that metabolic depletion triggered a compensatory response in the host at the transcriptional level, implying that the transcriptional activation of sugar synthesis is a feedback mechanism driven by sugar depletion. These changes may be associated with *C. ribicola* infection. In contrast to previous reports on the direct host–pathogen competition for sugars via sugar transporters [28], our study suggests that, following infection by *C. ribicola*, the host may enhance sugar biosynthesis in source organs. Although T6P–Tre-mediated sugar metabolic reprogramming was activated, sugar depletion still occurred in *C. ribicola* infected tissues.

The total sugar depletion in Korean pines correlated significantly with *C. ribicola* infection. Although host defense responses may contribute to sugar consumption, the strong positive correlation between the total sugar depletion and fungal DNA (Pearson’s *r* = 0.8319, *p* < 0.001) strongly suggests that rust colonization may be a major contributor to the extensive sugar loss in the blister area. A similar pattern was observed in susceptible wheat following *Puccinia triticina* inoculation, where Glu, total reducing sugars, and Suc declined by over 50% [29]. Rust fungi depend on host-derived sugars for survival and reproduction, directly channeling these carbohydrates into spore formation. Conversely, restricting the sugar supply effectively curtails fungal growth and sporulation [30]. This interpretation is further supported by the observation that healthy boles, which are the exclusive sites of blister formation, naturally harbor high sugar levels, suggesting that this local abundance of sugar may create a permissive environment for pathogen establishment. This highlights the critical reliance of obligate biotrophic pathogens on host carbon resources, and underpins our study’s rationale for managing KPBR via sugar metabolic intervention. The current data reveal a strong correlation but cannot establish a direct causal relationship between host sugar depletion and fungal sugar uptake. Further direct evidence will be acquired using isotope tracing experiments to unequivocally demonstrate sugar uptake by *C. ribicola* from Korean pine. This will definitively confirm that sugar is a key aspect of the host–pathogen interaction. It is worth noting that the transport of these sugars from the host to rust fungi may be mediated by transport proteins [31]. This study identified that the transport protein *Pkmst6* was significantly upregulated and showed a significant correlation with several sugars. However, further research is needed to determine whether *Pkmst6* is involved in the sugar efflux.

As a central regulator that adjusts resource allocation, T6P was involved in regulating the sustained synthesis of sugars in the blister area of Korean pine. Although sugar depletion was observed in infected Korean pine, more sugar synthesis genes were activated while degradation genes were suppressed, suggesting that the host exhibits a compensatory response to metabolic sugar depletion. This reprogramming may involve pathogen-induced T6P accumulation. As a central regulator of sugar metabolism, T6P influences sucrose allocation, energy distribution, and developmental progression, acting as an essential metabolic checkpoint to fine-tune plant energy management and adaptive growth across diverse developmental and environmental contexts [32,33]. In this study, T6P was significantly correlated with the reprogramming of sugar metabolism in Korean pine, as evidenced by its direct correlation with six upregulated synthesis genes and 10 downregulated degradation genes, as well as other forms of regulation (Figure 6c, |*r*| > 0.8, *p* < 0.05). This suggests that the central regulatory mechanism of T6P, which balances growth and survival based on internal energy states and external environmental signals [34], may act as a crucial switch for energy metabolism regulation and may serve as a candidate target for regulating the host’s energy metabolism to control KPBR. Functional validation is required before this can be considered a practical target.

Disruption of TPP function blocks the conversion of T6P into Tre, resulting in T6P accumulation and reduced Tre levels. This can modulate plant sugar metabolism and photosynthesis [35]. TPP may therefore serve as a new target for regulating T6P, indirectly influencing plant sugar metabolism by modulating the conversion between T6P and Tre. During the interaction between Korean pine and *C. ribicola*, T6P accumulation may result from *C. ribicola* interfering with the expression of *Pktpp* genes, thereby inhibiting T6P-to-Tre conversion. Consistent with this study, several *SlTPS* genes and the *SlTPP2* gene are specifically induced upon infection by *Botrytis cinerea* and *Pseudomonas syringae*. Silencing these genes alters Tre levels, suggesting their involvement in regulating Tre metabolism, including the T6P metabolic axis [36]. Previous reports have shown that pathogens can also manipulate host sugar metabolism via TPS-mediated T6P synthesis [37]. *Ralstonia solanacearum* utilizes its Type III secretion system to inject the effector protein RipTPS (possessing TPS activity) into plant cells, which is capable of synthesizing T6P directly within plant cells, thereby disrupting the host’s metabolism [38]. In addition to modulating host T6P levels via TPS activity, it also acts directly on the SnRK1α subunit, perturbing the T6P-SnRK1 interaction and thereby modulating host sugar metabolism. *Ustilago maydis* secretes the effector Hap1, which targets the SnRK1α subunit to prevent inhibition by high T6P levels. Therefore, it disrupts the T6P-SnRK1 interaction and enhances host starch metabolism to support fungal proliferation [39]. This study suggests that *Pktpp* may be a regulatory factor that regulates T6P and may modulate metabolism during the Korean pine–*C. ribicola* interaction. However, further verification is required to determine whether this regulatory process involves the pathogen.

It is demonstrated that *C. ribicola* infection causes significant disruption to sugar metabolism in Korean pine, notably affecting the T6P–Tre module. This suggests that pathogens not only suppress plant immunity but also act as metabolic engineers to secure host-derived carbohydrates [40,41,42]. Pathogens interfere with host metabolism and acquire nutrients through diverse strategies, including manipulating sugar transport, enhancing host metabolite biosynthesis, de novo synthesizing of pathogen-specific nutrients within host cells, and hijacking host developmental programs to release sugars. These processes are primarily orchestrated by effector proteins [40,43,44]. Despite the fundamental importance of sugar metabolism in host–pathogen interactions, the role of *C. ribicola* effectors in modulating host sugar metabolism remains critically understudied. Candidate effectors have been identified in *C. ribicola* through the functional characterization of Cri-9402 as a secreted effector protein of this pathogen [45,46]. These findings provide a compelling rationale for investigating whether T6P–Tre-centered reprogramming, as observed in this study, is actively driven by *C. ribicola* effectors. In subsequent work, in-depth research will be conducted into how *C. ribicola* utilizes effectors to regulate the TPP and T6P–Tre modules.

Although this study obtained preliminary results in the host, several limitations remain. The isotope tracing, metabolic flux analysis, and functional validation have not yet been performed. Therefore, the current data cannot determine the origin and fate of the related sugars. Second, the omics analysis included only three independent biological replicates per group (*n* = 3), which limits the assessment of biological variation, weaker effects, and different tree ages or disease stages. Although the results from independent-sample RT-qPCR were consistent, they can only support the expression trends of the tested genes and cannot replace expanded omics samples. In addition, more in-depth validation of multiple sugar-degrading enzyme activities would provide stronger support for the finding that host degrading enzymes were inhibited. Future studies will elucidate sugar metabolic changes on the pathogen side and further clarify sugar flux.

This study reveals that *C. ribicola* infection induces sugar redistribution in the blister area of Korean pine, with the T6P–Tre module functioning as a key metabolic node. The expressions of *Pktpp* genes were downregulated, which was associated with inhibited T6P-to-Tre conversion, promoting T6P accumulation and Tre depletion. This occurred alongside metabolic reprogramming characterized by sustained activation of sugar synthesis and inhibition of sugar degradation in the host. Beyond these mechanistic implications, the central role of *Pktpp* and the T6P–Tre module in sugar redistribution suggests a potential avenue for disease control. Future strategies should focus on modulating the T6P–Tre balance through *Pktpp* to restrict sugar availability to *C. ribicola*. Once the sugar supply is blocked, *C. ribicola* would be unable to accumulate sufficient hyphal biomass to reach the sporulation threshold, thereby inhibiting aeciospore production and interrupting the pathogen’s life cycle before alternate host infection, preventing a new disease from developing. By targeting sugar metabolism rather than directly killing the pathogen [13,42], this strategy avoids imposing fungicidal selection pressure and may therefore reduce the risk of resistance evolution. This offers a promising new avenue for the sustainable management of forest diseases.

## 4. Materials and Methods

### 4.1. Plant Materials and Sampling

From 2024 to 2025, three permanent plots were established in a 15-year-old planted stand of Korean pine in Benxi Manchu Autonomous County, Benxi City, Liaoning Province, China (41°14′ N, 124°12′ E). A total of 356 diseased trees were continuously monitored. Detailed information on the collected samples is provided in Table S3, and samples were deposited at the Key Laboratory of Alien Forest Pest Detection and Control, Heilongjiang Province, China. Based on the spatial distribution of KPBR (see Section 2.1 and Section 2.2), each diseased tree was divided into two areas: the blister area, meaning the portion of the bole 150 cm below the ground, exhibiting mature or developing aecia that produce yellow aeciospores, and the non-blister area, meaning the third whorl branches, with no visible aecia. Control samples were collected from healthy Korean pine (CK) at the corresponding locations and designated as “healthy bole” and “healthy branch”, respectively. The inner bark and phloem tissues were aseptically collected from each area. Some samples were immediately rapidly frozen in liquid nitrogen and stored at −80 °C for molecular and physiological analysis. Another part was fixed in FAA for histological observation. Three biological duplicate samples were collected from each area, each from a different single tree.

### 4.2. Pathogen Identification

The morphology of aeciospores was examined and photographed under a light microscope. Genomic DNA was extracted from the aeciospores using the CTAB method. The ITS and LSU regions of the ribosomal RNA were amplified using the primers listed in Table 1. PCR products were sequenced and retrieved using the BLAST in the NCBI database (https://blast.ncbi.nlm.nih.gov/Blast.cgi). The sequences were aligned using MEGA, and the maximum likelihood (ML) phylogenetic tree was constructed with *Ravenelia evansii* and *R. macowaniana* as the outgroups. The branch support rate was evaluated with 1000 bootstrap replicates.

### 4.3. Histochemical Staining and Fungal Colonization Assessment

Tissues fixed with FAA were embedded in paraffin and sectioned into 8-μm-thick sections, which were stained with Trypan blue–lactophenol solution to observe fungal hyphae. Based on hyphal density and distribution, colonization was scored on a scale of 0 to 4: 0, no hyphae; 1, very sparse; 2, locally sparse; 3, dense, locally bundle; 4, extremely dense, forming extensive networks. The average colonization level was calculated for each area.

### 4.4. Determination of Relative C. ribicola DNA Level by qPCR

The relative *C. ribicola* DNA level was quantified using qPCR [47] with primers specific to the ITS of *C. ribicola* (Cr-gp-F/R, Table 2) and normalized using the ITS of Korean pine (Pinus-ITS-F/R) as an internal reference. Reactions were performed with the GoTaq 2-Step RT-qPCR System (Promega, Madison, WI, USA) on a Bio-Rad iCycler iQ5 system (Bio-Rad, Hercules, CA, USA). Three biological replicates and two technical replicates were used for each sample. The relative fungal DNA accumulation was calculated using the 2^−ΔCq^ method.

### 4.5. Untargeted Metabolomics Profiling

To elucidate metabolic and transcriptional reprogramming in Korean pine upon *C. ribicola* infection, this study focused exclusively on the host side. Untargeted metabolomics analysis was performed on three biological replicates for each of the four tissue areas (blister area, non-blister area, healthy bole, and healthy branch). Metabolites were extracted and analyzed using an LC-30A ultra-high-performance liquid chromatography system (Shimadzu, Kyoto, Japan) coupled with tandem mass spectrometry (LC-MS/MS). DAMs were identified using fold-change (FC > 1.5 or <0.667) and *p* < 0.05 as screening criteria. Soluble sugars were identified from the total metabolite library based on the HMDB and Kyoto Encyclopedia of Genes and Genomes (KEGG). Before performing multivariate analysis, data were log-transformed to base 10 and Pareto-scaled.

### 4.6. Total Sugar Content Determination

Total sugar content was determined using the 3,5-dinitrosalicylic acid (DNS) colorimetric method (G0503W assay kit, Geruisi, Suzhou, China). The tissues were hydrolyzed with acid to release reducing sugars, which react with DNS to form colored amino compounds; the content was determined by measuring the absorbance at 500 nm. The total sugar content (mg/g fresh weight) was calculated based on the Glu standard curve.

### 4.7. Transcriptome Sequencing and Bioinformatics

The total RNA was extracted from three biological replicates for each tissue region area using the TRIzol reagent (Invitrogen, Carlsbad, CA, USA). RNA integrity was verified on an Agilent 2100 Bioanalyzer (Agilent Technologies, Santa Clara, CA, USA), and cDNA libraries were constructed and sequenced on the Illumina NovaSeq 6000 platform (Illumina, San Diego, CA, USA). The raw reads were quality-filtered using fastp v0.23.2 and aligned to the Korean pine reference genome using HISAT2 v2.2.1. Transcript abundance was quantified as the count matrix with StringTie v2.2.1, and DEGs were identified with DESeq2 (|Log_2_FC| > 1, *p* < 0.05).

Based on the KEGG ortholog and CAZy family annotations, soluble sugar-related genes were classified into three functional modules: synthesis, degradation and transport. GO and KEGG enrichment analyses were performed to characterize biological processes and metabolic pathways.

### 4.8. Targeted Glycomics and RT-qPCR Validation

Using targeted glycomics, ultra-high-performance liquid chromatography-triple quadrupole mass spectrometry (UHPLC-QqQ-MS/MS) was employed to perform absolute quantitative analysis of 32 glycometabolites in the same four tissue groups (three biological replicates per group). A calibration curve was constructed using sugar standards (Sigma-Aldrich, St. Louis, MO, USA), and the steps for metabolite extraction, derivatization, and multiple MRM transitions followed previously published protocols. Data acquisition and quantitative analysis were performed using MassLynx v4.2 (Waters, Milford, MA, USA). RT-qPCR was performed to validate the expression of Tre metabolism genes using the GoTaq 2-Step RT-qPCR System on a Bio-Rad iCycler iQ5 system. The *18S rRNA* and *Actin* genes were used as internal reference genes. Each sample was run in triplicate.

### 4.9. Starch Content Determination

Starch content was determined using the anthrone colorimetric method (G0507W assay kit, Geruisi, Suzhou, China). The tissues were hydrolyzed with acid to release Glu, which reacts with anthrone to form a colored complex. The content was determined by measuring the absorbance at 620 nm. The starch content (mg/g fresh weight) was calculated based on the Glu standard curve.

### 4.10. Enzyme Activity Assays

TPS, TPP and TRE activities were determined using commercial assay kits (G0556W, G0582W, and G0554W, respectively; Geruisi, Suzhou, China). The absorbance was measured at 340 nm for TPS, 520 nm for TPP, and 510 nm for TRE. TPS activity was expressed as nmol/min/g fresh weight, and TPP and TRE activities were expressed as μg/h/g fresh weight. All procedures were performed according to the manufacturer’s instructions.

### 4.11. Statistical Analysis

All statistical analyses were performed using R v4.3.2. Between-group differences in metabolomic and transcriptomic features were evaluated via permutation-based multivariate analysis of variance (PERMANOVA, 999 permutations) using the vegan package v2.6-4. Principal component analysis (PCA) was performed on Pareto-scaled data. Pairwise comparisons were made for individual metabolites, carbohydrates, and gene expression levels, using either Welch’s *t*-test or the Mann–Whitney U test as appropriate; a *p*-value of <0.05 was considered statistically significant. Pearson’s correlation was used to assess the correlations of relative *C. ribicola* DNA levels and total sugar contents and consumption. Spearman’s rank correlation was used to evaluate the correlations between T6P level and the expression of genes involved in sugar synthesis and degradation, and the results were visualized using Cytoscape 3.10.3. The effect size was expressed as Cohen’s *d*.

## 5. Conclusions

This study reveals that infected Korean pine undergoes severe sugar depletion in the blister area. This occurs alongside T6P–Tre-centered reprogramming of sugar metabolism. In the blister area of infected Korean pines, host sugars are extensively depleted to meet the high carbon demand of *C. ribicola* during aecia formation. In response to this sugar depletion, the host initiates transcriptional reprogramming of sugar metabolism, a process described by the “synthesis–accumulation decoupling” model. This reprogramming is characterized by the suppression of *Pktpp* expression, which blocks the conversion of T6P into Tre, thereby leading to T6P accumulation. Elevated T6P acts as a signal to promote sugar biosynthesis while inhibiting sugar degradation. The *Pktpp*-mediated T6P–Tre module could be a candidate target for regulating host sugar allocation and limiting sugar availability to the pathogen, potentially offering a new strategy for KPBR control.

## Figures and Tables

**Table 1:** Primers used for molecular identification of the pathogen.

Gene	Primer Name	Primer Sequence
ITS	ITS4	TCCTCCGCTTATTGATATGC
	ITS5	GGAAGTAAAAGTCGTAACAAGG
LSU	LROR	ACCCGCTGAACTTAAGC
	LR5	TCCTGAGGGAAACTTCG

**Table 2:** Primers used for qPCR quantification of relative *C. ribicola* DNA level.

Gene	Primer Name	Primer Sequence
*C. ribicola* ITS	Cr-gp-F	GGTTAGGAAGCTATTTATCTGAAACA
	Cr-gp-R	GAGTGTTGCTGTCATTGGCTC
*P. koraiensis* ITS	Pinus-ITS-F	TAACACACGGAAACGACTCTCG
	Pinus-ITS-R	GACGCCCAGACAGACGTG

## Data Availability

The genetic information in this study is available in the National Genomics Data Center (NGDC) database under accession number CRA050387 (https://ngdc.cncb.ac.cn/gsa, accessed on 29 September 2026). The raw metabolomics data are not publicly available and are available from the corresponding author upon reasonable request.

## Supplementary Materials

The following supporting information can be downloaded at: https://www.mdpi.com/article/10.3390/ijms27219441/s1.

## Abbreviations

The following abbreviations are used in this manuscript:

Tre	Trehalose
T6P	Trehalose-6-phosphate
M6P	Mannose 6-phosphate
R5PB	Barium D-ribose-5-phosphate
Glu	Glucose
Suc	Sucrose
Mal	Maltose
Raf	Raffinose
NSCs	Non-structural carbohydrates
SCs	Structural carbohydrates
PCA	Principal component analysis
DAMs	Differentially accumulated metabolites
DEGs	Differentially expressed genes
GO	Gene Ontology
KEGG	Kyoto Encyclopedia of Genes and Genomes
RT-qPCR	Real-time quantitative PCR
FBA	Fructose bisphosphate aldolase
RafS	Raffinose synthase
SuSy	Sucrose synthase
GH	Glycosyl hydrolase
AMY	Alpha amylase
