# Supplementary material for: Sugar Depletion in Korean Pine Infected with Cronartium ribicola, Alongside Metabolic Reprogramming by the T6P–Tre Signaling Module
Source: Int J Mol Sci. 2026 Oct 23;27(21):9441. doi: 10.3390/ijms27219441 (PMC13762924; doi:10.3390/ijms27219441)
Supplement: Supplementary file 1 [file ijms-27-09441-s001.zip › ijms-4585462-supplementary.pdf]

***Supplementary Material***

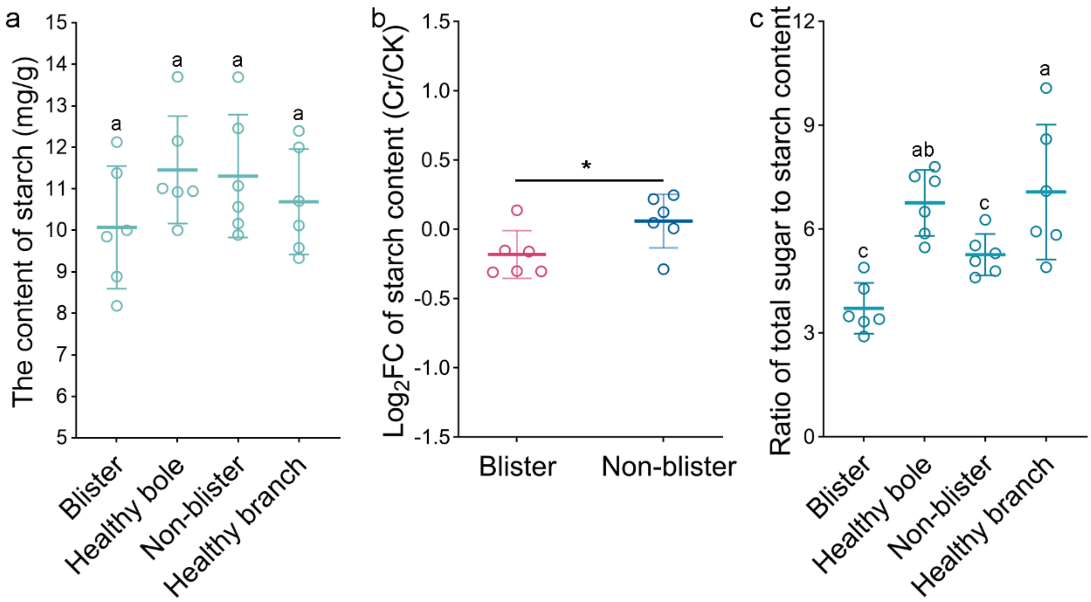


**Figure S1.** Relative starch content in different tissues: (**a**) the determination of starch content; (**b**) relative starch content; (**c**) ratio of total sugar to starch content.

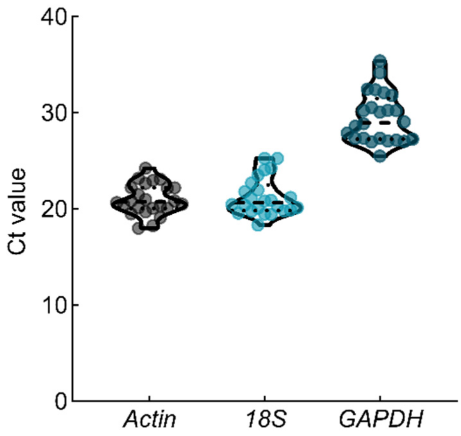


**Figure S2.** The Ct values of three reference genes in blister area, non-blister area, healthy bole and healthy branch separately collected in 2024 and 2025.

**Table S1.** Transcriptome-based interaction effects of Tre metabolism genes between blister and non-blister tissues in response to *Cronartium ribicola* infection.

| Gene | Interaction $Log_2FC$ | SE | 95% CI Lower |
|---|---|---|---|
| Cluster-15913.15 | -2.23 | 0.39 | -3.13, -1.32 |
| Cluster-113777.4 | -1.53 | 0.62 | -2.96, -0.10 |
| Cluster-113777.1 | -1.03 | 0.25 | -1.62, -0.44 |
| Cluster-117942.16088 | 1.10 | 0.79 | -0.73, 2.93 |
| Cluster-112153.3 | 1.18 | 0.62 | -0.25, 2.61 |
| Cluster-117942.15636 | 0.04 | 0.82 | -1.86, 1.93 |
| Cluster-123895.256 | 0.24 | 0.73 | -1.43, 1.91 |
| Cluster-124487.1 | 1.25 | 0.20 | 0.79, 1.70 |
| Cluster-117942.15629 | -0.24 | 0.91 | -2.33, 1.86 |
| Cluster-15913.16 | -0.79 | 0.76 | -2.55, 0.97 |
| Cluster-123895.283 | -0.48 | 0.79 | -2.30, 1.34 |
| Cluster-101135.11 | -0.53 | 0.48 | -1.65, 0.58 |
| Cluster-113777.3 | -0.08 | 1.00 | -2.38, 2.21 |
| Cluster-112153.4 | -0.71 | 0.30 | -1.39, -0.03 |
| Cluster-108757.0 | -0.36 | 0.11 | -0.62, -0.10 |
| Cluster-108716.0 | -1.60 | 0.55 | -2.87, -0.33 |
| Cluster-129996.10 | -1.11 | 0.38 | -1.99, -0.23 |
| Cluster-129996.7 | -2.17 | 0.94 | -4.33, -0.01 |
| Cluster-57865.1 | 3.13 | 1.09 | 0.62, 5.63 |
| Cluster-30363.0 | 1.67 | 0.98 | -0.60, 3.94 |
| Cluster-36186.0 | 1.28 | 0.68 | -0.30, 2.85 |
| Cluster-129996.1 | -0.31 | 0.45 | -1.34, 0.73 |

**Note:** For the 22 Tre metabolism genes, the overall error standard deviation ($\sigma$) was 0.5655. The statistical power was 0.8894 when the minimum acceptable |Interaction $Log_2FC$| was set at 1.0.

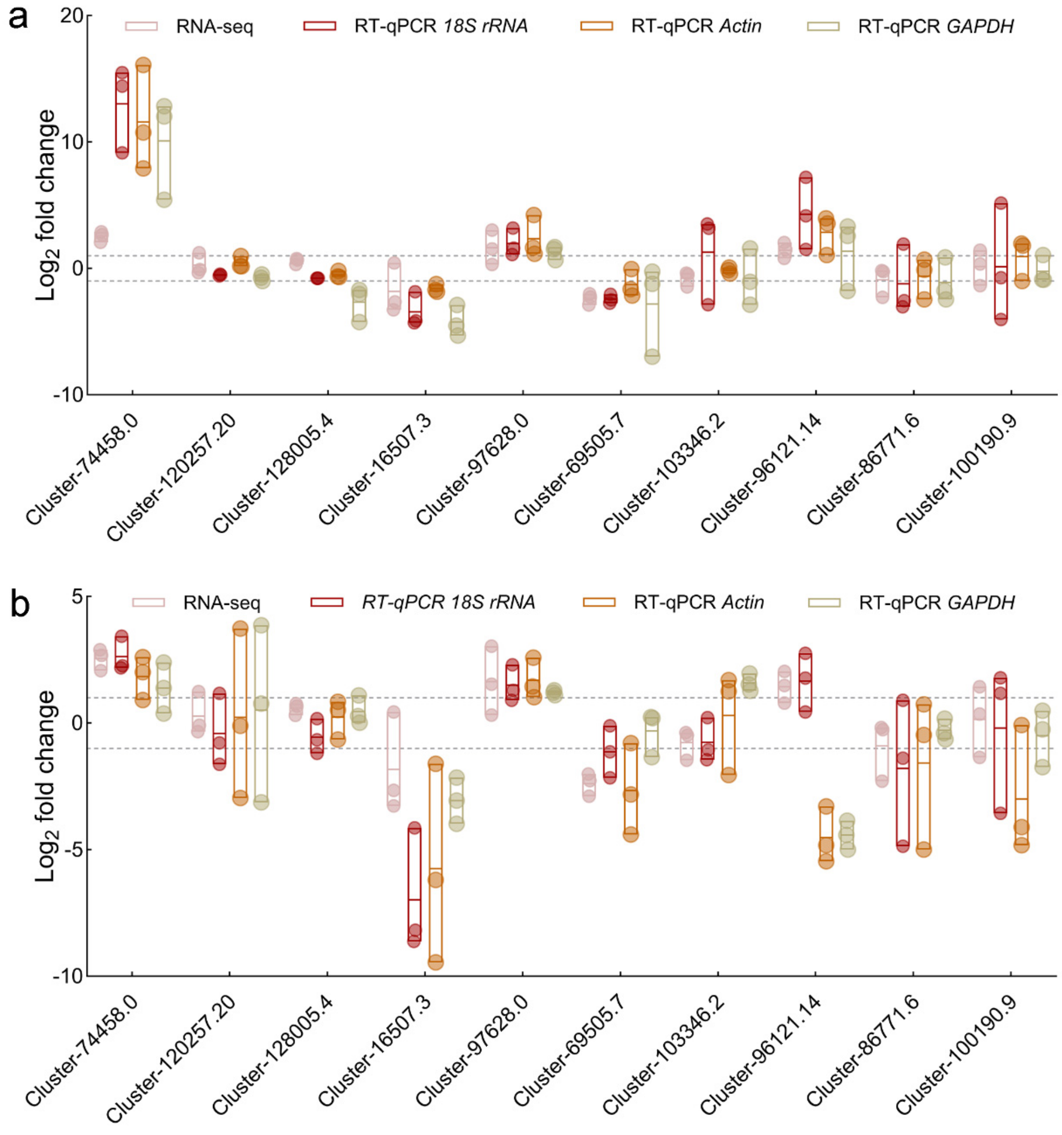


**Figure S3.** RT-qPCR of the randomly selected ten genes to validate the transcriptome data: (**a**) samples collected in 2024; (**b**) samples collected in 2025.

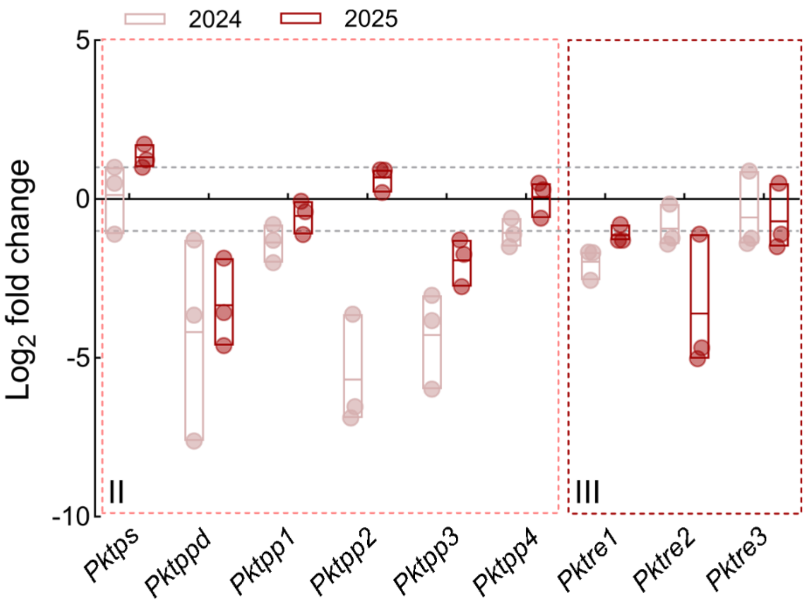


**Figure S4.** RT-qPCR of the Tre biosynthetic genes and trehalase genes using *GAPDH* as a reference gene.

**Table S2.** RT-qPCR validation of interaction effects for key Tre metabolism genes between blister and non-blister tissues under *C. ribicola* infection.

| Gene | Interaction $Log_2FC$ | SE | 95% CI |
|---|---|---|---|
| *Pktps* | 1.74 | 0.43 | 0.64, 2.84 |
| *Pktppd* | -3.46 | 0.37 | -4.42, -2.51 |
| *Pktpp1* | -1.33 | 0.19 | -1.82, -0.85 |
| *Pktpp2* | -1.97 | 1.00 | -4.55, 0.60 |
| *Pktpp3* | -2.02 | 0.53 | -3.38, -0.65 |
| *Pktpp4* | -0.99 | 0.24 | -1.62, -0.37 |
| *Pktre1* | -2.90 | 0.63 | -4.51, -1.29 |
| *Pktre2* | -2.80 | 0.44 | -3.93, -1.67 |
| *Pktre3* | -1.19 | 0.21 | -1.72, -0.66 |

**Note:** For the nine Tre metabolism genes validated by RT-qPCR, the overall error standard deviation (σ) was 1.0475. The statistical power was 0.9264 when the minimum acceptable |Interaction $Log_2FC$| was set at 1.0.

**Table S3.** Sample collection information.

| Sample | Tree Age (year) | Sampling Time | Collection Site | Infection Duration (year) |
|---|---|---|---|---|
| Blister | 15 | May 2024 | Benxi City, Liaoning Province, China (41°14′ N, 124°12′ E) | 3 |
| Non-blister | 15 | May 2024 | Benxi City, Liaoning Province, China (41°14′ N, 124°12′ E) | 3 |
| Healthy bole | 15 | May 2024 | Benxi City, Liaoning Province, China (41°14′ N, 124°12′ E) | - |
| Healthy branch | 15 | May 2024 | Benxi City, Liaoning Province, China (41°14′ N, 124°12′ E) | - |
| Blister | 15 | May 2025 | Benxi City, Liaoning Province, China (41°14′ N, 124°12′ E) | 3 |
| Non-blister | 15 | May 2025 | Benxi City, Liaoning Province, China (41°14′ N, 124°12′ E) | 3 |
| Healthy bole | 15 | May 2025 | Benxi City, Liaoning Province, China (41°14′ N, 124°12′ E) | - |
| Healthy branch | 15 | May 2025 | Benxi City, Liaoning Province, China (41°14′ N, 124°12′ E) | - |
